# Physiological feedback technology for real-time emotion regulation: a systematic review

**DOI:** 10.3389/fpsyg.2023.1182667

**Published:** 2023-05-12

**Authors:** Yifan Sun, Tian Lu, Xuanyi Wang, Wanlin Chen, Shulin Chen, Hang Chen, Jing Zheng

**Affiliations:** ^1^College of Biomedical Engineering and Instrument Science, Zhejiang University, Hangzhou, Zhejiang, China; ^2^Department of Psychological and Behavioral Science, Zhejiang University, Hangzhou, Zhejiang, China

**Keywords:** physiological feedback, emotion, emotion regulation, systematic review, mental health

## Abstract

**Background:**

Previous studies suggest that physiological feedback can be an effective method for emotion regulation (ER). However, studies on the specific effects of physiological feedback have shown conflicting results due to inconsistencies in study designs. Therefore, we present this systematic review to further validate the effectiveness of physiological feedback for ER, clarify its specific effects, as well as summarize the factors that influence its effectiveness.

**Method:**

This systematic review following PRISMA guidelines covers all studies using physiological feedback in emotions. A literature search was conducted in Web of Science, PubMed, PsychINFO, China National Knowledge Infrastructure, and WANFANG DATA. And a standardized quality assessment was performed.

**Results:**

We identified 27 relevant articles (25 studies), and the majority of these studies showed a significant regulatory effect of physiological feedback on different emotions. The feedback's content, explanation, authenticity, real-time capability, and modality were the key factors that influenced its effects, and this technology will achieve its optimal ER effect when these factors are considered comprehensively.

**Conclusions:**

These findings further confirmed the effectiveness of physiological feedback as an ER method, as well as providing key factors that should be addressed in its application. Meanwhile, due to the limitations of these studies, more well-designed studies are still needed.

## 1. Introduction

Emotions are an integral part of our lives. All individuals feel and experience emotions every day (Rolston and Lloyd-Richardson, 2017). In most cases, emotions involve a mental state that arises spontaneously rather than through conscious effort (Shu et al., 2018). Our desire to control emotions, rather than experience them passively, has contributed to the emergence of the field of emotion regulation (ER) (Gross, 2015). ER involves an attempt to influence emotions, including up-regulation and downregulation of positive and negative emotions according to the corresponding regulation goals (McRae and Gross, 2020).

ER is crucial for psychological well-being and overall functioning (Gross, 2015). Effective ER enables individuals to better cope with challenging situations, adapt to changes, and maintain psychological well-being (Aldao et al., 2010). And difficulties in ER have been linked to a range of mental health issues, including increased risk of mood disorders, maladaptive coping strategies, and interpersonal conflicts (Gratz and Roemer, 2003).

The application of digital technologies in the ER field has gained increasing attention in recent years, with emerging evidence suggesting their potential effectiveness (Wadley et al., 2020; Bettis et al., 2022; Drigas et al., 2022; Mitsea et al., 2023). Virtual reality technology integrated computer graphics with sensory experiences to create an environment that mirrors the real-world, and enables users to interact and engage in a variety of real-world scenarios, providing a highly immersive and interactive user experience (Bettis et al., 2022). In recent years, virtual reality technology has been used in various emotional contexts and becomes a viable tool for helping users regulate their emotions (Colombo et al., 2019; Macey et al., 2022). Biofeedback training technology use and monitor different physiological factors to provide users with awareness and insight into their physiological changes, helping them better control those changes, and it has been shown to be one of the useful ways to regulate emotions (Colombo et al., 2019; Kennedy and Parker, 2019; ter Harmsel et al., 2021). Mindfulness-based technology is becoming increasingly used for ER as well. Mindfulness can be defined as the practice of consciously and openly attending to one's present-moment experience with awareness (Creswell, 2017). And in recent years, mindfulness-based emotion regulation techniques have gained attention as effective interventions for enhancing emotion regulation skills and promoting mental well-being (Surawy et al., 2005; Mitsea et al., 2023). There are other novel technologies and approaches that show the potential to provide emotional management support, including smart-home technology, online cognitive-behavioral therapy programs, and artificial intelligence chatbots (Andrews et al., 2018; Boucher et al., 2021; Bettis et al., 2022).

However, some studies have pointed out that the effectiveness of ER strategies depends on the emotional context in which they are used (Kobylińska and Kusev, 2019), suggesting that the effectiveness of ER strategies and their corresponding technologies tend to be different when applied to different emotional situations. For example, most of the prevailing ER technologies, including those mentioned above, are probably incapable of achieving effective ER for anxiety during a speech. This is because, for this emotional situation, the ER technology not only needs to be effective rapidly to relieve anxiety on time but also requires less attention and effort from the user to avoid disrupting the ongoing presentation (Adams et al., 2015). Therefore, in many emotional situations such as those that require effective real-time intervention without interference with ongoing tasks, there is still a need for an emotion management technology in addition to the currently available technologies.

As an ER technology capable of meeting these requirements, physiological feedback has been proven to be effective in many studies (Crucian et al., 2000; Chittaro, 2014; Costa et al., 2016; Azevedo et al., 2017). Physiological feedback captures physiological responses of the human body and provides real-time feedback to users, enabling them to enhance their awareness of their physiological states. Unlike traditional biofeedback technology, physiological feedback simply delivers physiological signals to users without requiring their complete focus or active control of these signals (Yucha and Montgomery, 2008; Costa et al., 2016).

Precisely because physiological feedback does not require users to focus entirely on its content, this technology has minimal attentional demands and does not impose a heavy cognitive burden on the individual (Costa et al., 2016). This attribute endows physiological feedback with the potential to be employed in diverse scenarios, including those where users may have limited attentional resources or need to multitask (e.g., everyday life, workplaces, schools, and clinical settings), it also allows this technology accessible to a wide range of populations, including those with limited cognitive or emotional capacities (e.g., autism spectrum disorder, attention deficit hyperactivity disorder, social anxiety disorder). Moreover, integrating physiological feedback with other established technologies or interventions, such as mindfulness-based interventions or psychotherapy, holds promise as a viable approach to enhance their efficacy in the field of ER (Bettis et al., 2022).

Despite the promising potential of physiological feedback, it is difficult to reach a consensus among studies about the effectiveness of this technology for ER (Borkovec et al., 1974; Misovich, 1974; Parkinson and Colgan, 1988; Telch et al., 2000; Azevedo et al., 2017; Dey et al., 2018). This is probably because relevant studies used different target emotions, different feedback modalities (e.g., heart rate feedback and galvanic skin response feedback), and inconsistent feedback conditions (e.g., true feedback and sham feedback) (Menyhart and Gleary, 1986; Tajadura-Jimenez et al., 2008; Makkar and Grisham, 2013; Azevedo et al., 2017; Dey et al., 2018; Ehlers et al., 2021). Therefore, there is an urgent need for a systematic review of this technology to clarify its effectiveness when targeting different emotions, using different feedback modalities, and setting different feedback conditions.

The main objective of this study was to perform the first systematic review that focused on the use of physiological feedback to regulate ongoing emotions. Through a comprehensive analysis of the relevant studies, we provide an overview of this technology as an ER intervention to clarify its effectiveness and summarize the factors that influence its effects as well as make recommendations for its implementation in further research and real-life practice.

The rest of this paper is organized into four sections. Section 2 details the methodology used in conducting the systematic review, it includes information on the search strategy used to identify relevant studies, inclusion and exclusion criteria for study selection, data extraction methods, and quality assessment. Section 3 presents the findings of this review, including the results of the study selection, overall features of the included studies, and key findings from each study. In Section 4, we interpret the results of this review and discuss the effects of physiological feedback technology and the factors that influence its effectiveness. We also point out the limitations of these studies and make suggestions for future research in this section. Finally, we conclude in Section 5.

## 2. Materials and methods

### 2.1. Search strategy

This systematic review was designed following the Preferred Reporting Items for Systematic Reviews and Meta-Analysis Protocols (PRISMA) statement (Page et al., 2021). A systematic search was performed using the following electronic databases: Web of Science, PubMed, PsychINFO, China National Knowledge Infrastructure, and WANFANG DATA. Among these databases, the Web of Science, PubMed, and PsychINFO were used for English articles, whereas the China National Knowledge Infrastructure and WANFANG DATA were used for Chinese articles. The search was conducted in September 2022, and there were no limitations regarding the start of the search period.

According to the aim of this review, the two key concepts used for searching were “physiological feedback” and “emotion.” After several attempts, the concepts of “biofeedback,” “physiological feedback,” “heart rate feedback,” “tactile feedback,” “galvanic skin response feedback,” “respiratory feedback,” or “temperature feedback” were used in combination with “emotion” as search terms. The search strategy used the following syntax: Emotion AND (“biofeedback” OR “physiological feedback” OR “heart rate feedback” OR “tactile feedback” OR “galvanic skin response feedback” OR “respiratory feedback” OR “temperature feedback”).

### 2.2. Selection criteria

All articles identified by the search were selected according to the following inclusion and exclusion criteria. The inclusion criteria included studies (1) that were original and involved experiments; (2) that used emotion eliciting materials to elicit emotions; (3) that included participants of whom all or part received physiological feedback during emotion elicitation; and (4) that did not ask participants to try to change the feedback provided. The exclusion criteria included (1) review articles, (2) non-experimental studies, (3) non-specific emotional interventions, (4) studies with no physiological feedback included or biofeedback training, or (5) studies that were published in a foreign language.

### 2.3. Selection procedure

After the primary selection of papers by applying the search strings to the electronic databases, duplicate publications were excluded. Thereafter, titles and abstracts of studies were screened for eligibility, and the remaining candidate articles were screened using full-text versions according to pre-defined inclusion and exclusion criteria. In addition, we also found a suitable article through the bibliography of other articles, which was added to the eligibility evaluation stage of the selection process. During this process, related studies cited in the articles were progressively added to the final included articles.

### 2.4. Data extraction

For the studies included in the review, a systematic data extraction procedure was used to determine the main characteristics of each article. Five categories of information were extracted from the studies:

(1) Participants: basic information about participants in the studies, including the population, sample size, mean age, age range, and gender ratio;
(2) Emotional intervention: emotion elicitation process of the studies, including the emotion (type) involved in the experiment and its elicitation materials;
(3) Physiological feedback: features of physiological feedback, including presentation form of the feedback, experimental groups, feedback conditions, the authenticity of the feedback (i.e., whether the feedback is based on the actual physiological state of the subject), and the real-time capability of the feedback (i.e., whether the feedback changes according to the real-time physiological state of the subject);
(4) Groups and conditions: experimental groups and feedback conditions;
(5) Measures and results: evaluation methods used in the research and the corresponding results. Both qualitative and quantitative information were extracted (i.e., psychological and physiological measures and statistical significance).

### 2.5. Quality assessment

Study quality was assessed independently by two reviewers. As all included studies were cross-sectional, they were evaluated using the Quality Assessment Tool for Observational Cohort and Cross-sectional Studies (National Heart Lung and Blood Institute, 2021). And studies were rated as “Good,” “Fair,” or “Poor” quality according to the guidance given by this tool.

## 3. Results

### 3.1. Identified studies

**Figure 1:**
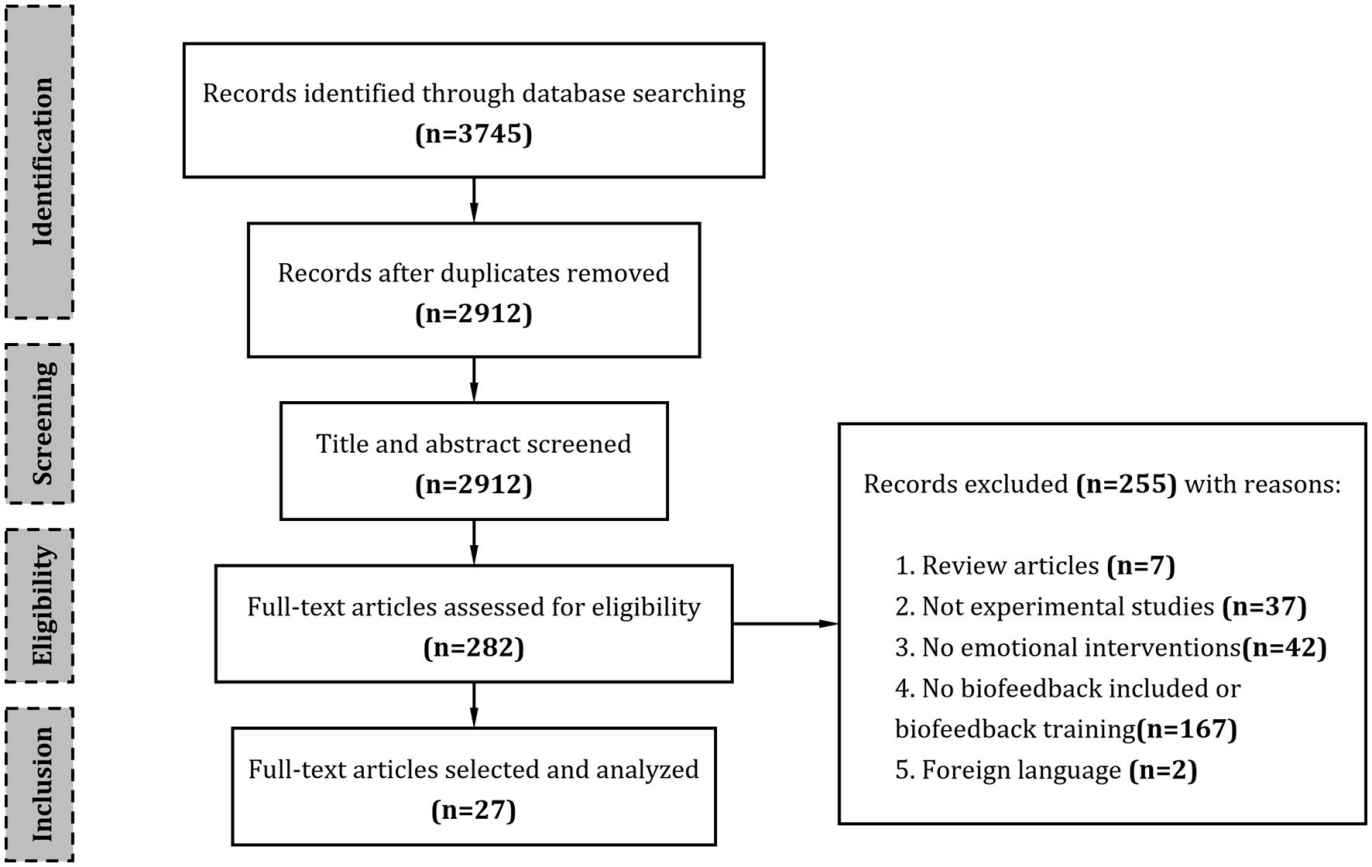
Flowchart of the study selection process.

Based on the information sources and search strategy mentioned above, the initial literature search yielded a total of 3,745 articles that were relevant to this review. Of those, 2,912 articles remained after duplicates were removed. After title and abstract screening, 282 articles remained. The full texts of these 282 articles were examined for eligibility based on the inclusion and exclusion criteria described above. Finally, a total of 27 articles (25 studies) were included in this review, and their information was extracted and summarized. An overview of the selection process is visualized in Figure 1.

### 3.2. Study quality

An assessment of study quality revealed that four studies were rated as “Good.” In these studies, the population was specified, the outcome measures were valid and reliable, and several confounding variables were considered. Nineteen studies were rated as “Fair,” indicating susceptibility to bias. Two studies were rated as “Poor,” indicating a significant risk of bias. These studies had unclear methods for recruitment, insufficient consideration of confounding variables or included self-reported diagnosis as the primary method for determining the effects of physiological feedback. A detailed assessment of the included studies' internal validity is presented in Appendix A.

### 3.3. Study characteristics

A total of 1,364 participants were included in the 27 articles (25 studies). Among these studies, two articles written by Hirschman (1975), Hirschman and Hawk (1978), each describing partial results of the same experiment, were grouped into one study to achieve a more accurate assessment. Similarly, the results obtained from two articles written by Chittaro (Chittaro, 2012, 2014) were also grouped into one study.

Of all the studies, 21 were conducted at universities and involved students as the main participants and 4 studies had no reference to their population in the article. The sample sizes ranged from 19 to 120, with those of most studies ranging from 30 to 60. Some of the studies did not report the age information of the subjects, while the majority of the studies were aimed at young people. Targeted emotions varied widely: 7 studies only on lust, 8 studies only on anxiety, 3 studies only on fear, 3 studies only on unpleasantness, 5 studies on one or more non-specific emotions (positive, neutral, and negative), and 1 study on five emotions (happiness, anxiety, fear, disgust, and sadness). The materials used to elicit these target emotions included relevant images (11), specific tasks (7), music or audio (3), videos (1), and virtual reality scenes (2).

The feedback modalities used in these studies included Heart Rate (HR) (22), Galvanic Skin Response (GSR) (4), pupil size (1), and physiological arousal state (1). The presentation form of feedback included auditory form (20), such as heartbeat sound, visual form (2), such as GSR readings, and haptic form (3), such as heartbeat vibration. As for the authenticity and real-time capability of the feedback, the feedback in 17 studies was neither authentic nor real-time, while the feedback in 1 study was authentic but not real-time, but in 7 studies, the feedback was authentic and real-time in at least one of the feedback conditions. The characteristics of participants, emotion intervention, and physiological feedback is presented in Table 1.

**Table 1:** Characteristics of participants, emotion intervention, and physiological feedback.

**Reference**	**Participants**		**Emotion intervention**		**Physiological feedback**			
	**Population**	**Number; age; gender**	**Emotion type**	**Intervention material**	**Modality**	**Presentation form**	**Authenticity**	**Real-time capability**
Azevedo et al. (2017)	NR	*N* = 52 Age: 26.4 ± 5.7 F|M: 32|20	Anxiety	A public speech task	HR	Haptic-heartbeat vibration	Based on real physiological status	Not real-time
Borkovec et al. (1974)	University students with speech-anxiety	*N* = 60 Age: NR Gender: NR	Anxiety	Three consecutive speeches tasks	HR	Auditory-heartbeat sound	Not based on real physiological status	Not real-time
Botto et al. (1974)	Introductory psychology students	*N* = 60 Age: NR F|M: 0|60	Lust	Images of seminude females	HR	Auditory-heartbeat sound	Not based on real physiological status	Not real-time
Chittaro (2012, 2014)	University students and others	*N* = 108 Age: 24.1 ± 3.2 F|M: 24|84	Anxiety	VE of being suddenly surrounded by smoke during a fire evacuation of a building.	HR	Auditory-heartbeat sound	Based on real physiological status	Real-time
Costa et al. (2016)	University students	*N* = 67 Age: (19-30) F|M: 43|24	Anxiety	A presentation task	HR	Haptic-heartbeat vibration	Based on real physiological status (only TH condition)	Real-time (only TH condition)
Dey et al. (2018)	University students, staff and others	*N* = 19 Age: 30.6 ± 7.1 (21–45) F|M: 2|17	Mixed emotions (happiness, anxiety, fear, disgust, sadness)	VE of a jungle safari with various animals moving through	HR	Auditory and haptic-heartbeat sound and heartbeat vibration	Based on real physiological status	Real-time
Ehlers et al. (2021)	NR	N = 48 Age: 25 ± 10 F|M: 40|8	Non-specific positive and negative emotions	Positive and negative sounds from iad-2	Exp1: pupil size Exp2: GSR	Exp1: visual-pupil size change animation Exp2: visual-scr changes waveform	Based on real physiological status	Real-time
Goldstein et al. (1972)	Introductory psychology students	*N* = 60 Age: NR Gender:NR	Lust	Images of nude females	HR	Auditory-heartbeat sound	Not based on real physiological status	Not real-time
Hirschman (1975); Hirschman and Hawk (1978)	University students and others	*N* = 48 Age: NR F|M: 48|0	Unpleasant	Images of people who died violently	HR	Auditory-heartbeat sound	Not based on real physiological status	Not real-time
Hirschman et al. (1977)	University students	*N* = 36 Age: NR F|M: 0|36	Lust	Images from playboy magazine	HR	Auditory-heartbeat sound	Not based on real physiological status	Not real-time
Makkar and Grisham (2013)	University students with high and low social anxiety	*N* = 80 Age: NR F|M: 48|31	Anxiety	A videotaped speech task	HR	Auditoryandvisual-beeping noise whose frequency match the HR and ECG waveform and hr number	Not based on real physiological status	Not real-time
Menyhart and Gleary (1986)	University students	*N* = 54 Age: NR Gender: NR	Fear	A task of walking toward the edge of a flat roof area 20 m above ground level	HR	Auditory-heartbeat sound	Not based on real physiological status	Not real-time
Misovich (1974)	Introductory psychology students	*N* = 44 Age: NR F|M: 0|44	Lust	Images of nude females	GSR	Visual-GSR readings	Not based on real physiological status	Not real-time
Pan et al. (2020)	University students	*N* = 36 Age: NR F|M: 21|15	Non-specific positive and neutral emotions	Positive and neutral music	HR	Haptic-heartbeat feel	Based on real physiological status	Real-time
Parkinson and Manstead (1986)	University students	*N* = 80 Age: (18–35) F|M: 40|40	Lust	Images of nude or seminude females	HR	Auditory-heartbeat sound	Not based on real physiological status	Not real-time
Parkinson and Colgan (1988)	University students	*N* = 80 Age: (18–35) F|M: 0|80	Non-specific positive and negative emotions	Images of animals (negative: various unpleasant insects, spiders, and snakes; positive: kittens and other “cute” animals)	GSR	Auditory-a tone whose changing loudness reflected GSR variation	Not based on real physiological status	Not real-time
Rohrmann et al. (1999)	University students and others	*N* = 60 Age: 24.85 ± 3.32 (18–33) F|M: 0|60	Anxiety	A public speech task	Physiological arousal state	Auditory-verbal comments	Not based on real physiological status	Not real-time
Shahidi and Baluch (1991)	NR	N = 120 Age: 20.5 (18–32) Gender:NR	Anxiety	A speech task in front of a camera	HR	Auditory-heartbeat sound	Not based on real physiological status	Not real-time
Stern et al. (1972)	University students	*N* = 60 Age: NR F|M: 0|60	Lust; unpleasant	Images from playboy magazine; images of people who were badly injured or dead because of car accidents	HR	Auditory-heartbeat sound	Not based on real physiological status	Not real-time
Tajadura-Jimenez et al. (2008)	NR	*N* = 24 Age: 24.4 ± 4.6 F|M: 6|18	Non-specific positive and negative emotion	Positive and negative images from IAPS (5 on a 9-point arousal scale, 3 and 7 on a 9-point valence scale for negative and positive images respectively)	HR	Auditory and haptic-heartbeat sound and heartbeat vibration	Not based on real physiological status	Not real-time
Telch et al. (2000)	University students with claustrophobic fear	*N* = 54 Age: 17.9 ± 0.6 F|M: 46|8	Fear	A task of exposure to a claustrophobic chamber	HR	Auditory-tones whose frequency reflected HR variation	Based on real physiological status (only TH condition)	Real-time (only TH condition)
Thornton and Hagan (1976)	NR	*N* = 30 Age: (19–27) Gender: NR	Unpleasant	Images of skin disease	HR	Auditory-heartbeat sound	Not based on real physiological status	Not real-time
Valins (1966)	Introductory psychology students	*N* = 60 Age: NR F|M: 0|60	Lust	Images of seminude females	HR	Auditory-heartbeat sound	Not based on real physiological status	Not real-time
Wang et al. (2020)	University students	*N* = 48 Age: 21.63 ± 1.35 (20-24) F|M: 12|12	Non-specific positive and neutral emotions	Positive and neutral music	HR	Auditory and haptic-heartbeat sound and heartbeat feel	Based on real physiological status (only TH condition)	Real-time (only TH condition)
Young et al. (1982)	University students with dentally anxious	*N* = 24 Age: NR F|M: 12|12	Anxiety	A video of dental stimulus	HR	Auditory-heartbeat sound	Not based on real physiological status	Not real-time

TH, True heartbeat feedback; GSR, Galvanic Skin Response; HR, Heart Rate; NR, not reported.

The experimental groups and feedback conditions were also recorded to facilitate the presentation and analysis of the results. All of the studies included at least one experimental condition using physiological feedback technology. Meanwhile, we named some common experimental conditions and represented them using the following abbreviations: Constant Heartbeat feedback (CH): heartbeat feedback with a constant frequency; Increasing Heartbeat feedback (IH): heartbeat feedback with an increasing frequency; Decreasing Heartbeat feedback (DH): heartbeat feedback with a decreasing frequency; True Heartbeat feedback (TH): heartbeat feedback with a frequency consistent with the participants' actual heartbeat; and No Feedback (NF): no feedback control condition.

The measurements included psychological measurements (24) and physiological measurements (18). The characteristics of groups and conditions, measurements, and results is presented in Table 2.

**Table 2:** Characteristics of groups and conditions, measurements, and results.

**Reference**	**Groups or conditions**	**Measurements and Results**			
		**Psychological measurements**	**Physiological measurements**	**Psychological results**	**Physiological results**
Azevedo et al. (2017)	CH (20% slower than baseline), NF	STAI-Y-1, BFNE, 7-point likert scales	HR, GSR	Anxiety level: CH condition was significantly lower than NF condition after speech preparation (*p* = 0.007)	SCL: CH condition was significantly lower than NF condition (p = 0.029)
Borkovec et al. (1974)	IH, CH, DH, IH (no explanation), NF	Brief anxiety questionnaire, observer's ratings of overt signs of anxiety, number of speech disfluencies	HR	Anxiety level: (1) no significant differences among the feedback conditions during the feedback speech; (2) DH and CH conditions were significantly lower than IH condition in posttest speech measures (*p* < 0.05)	HR: No significant main or interaction effects of the feedback conditions
Botto et al. (1974)	3 rates (CH, IH, DH) x 3 (HR group: explained as heartbeat sounds, ES group: explained as as extraneous sounds, ES-T-I group: explained as extraneous sounds and instruct to attend)	Image ratings about lust;	/	Lust level: within the HR group, (1) CH condition was significantly lower than IH condition (*p* = 0.02); (2) DH condition was significantly lower than IH condition (*p* = 0.05)	/
Chittaro (2012, 2014)	Health Bar condition: a horizontal green bar displayed the level of health of the user's avatar during the virtual experience FPS condition: exploited the aversive auditory and visual stimuli to indicate that the user's avatar is getting hurt bioFPS condition: was identical to the FPS condition except for the fact that heartbeat sound was controlled by the proposed biofeedback mechanism.	STAI	GSR	Anxiety level: bioFPS condition was higher than FPS condition, FPS condition was higher than health bar condition (significant difference between bioFPS and Health Bar, *p* < 0.05)	SCL: bioFPS condition was significantly higher than other two conditions (*p* < 0.05)
Costa et al. (2016)	NF, TH, 60 bpmCH, vibration (60 bpm without explanation)	STAI	/	Anxiety level: (1) 60 bpm CH condition was significantly lower than NF condition (*p* = 0.014); (2) 60 bpm CH condition was significantly lower than TH condition (*p* = 0.018)	/
Dey et al. (2018)	TH, +30%IH, +15%IH,−15%DH,−30%DH	PANAS, SAM	HR, GSR	No significant difference between the conditions on either positive affect (*p* = 0.17) or negative affect (*p* = 0.18), but five individual emotions were significantly influenced by the heart rate manipulation—interest, excitement, scariness, nervousness, and fear.	No significant difference among feedback conditions
Ehlers et al. (2021)	Exp1: NR Exp2: true feedback, strong sham feedback, weak sham feedback	exp1: SAM, MAIA	Exp1: pupil diameter Exp2: SCR	/	pupil diameter: no significant difference among feedback conditions SCR: true feedback condition was significantly higher than other two conditions (*p* < 0.05)
Goldstein et al. (1972)	IH, CH, NF	Image ratings about lust;	HR	Lust level: CH condition was significantly lower than IH condition (*p* < 0.001)	HR (HR's absolute deviation): CH condition was significantly lower than IH condition (*p* < 0.001)
Hirschman (1975); Hirschman and Hawk (1978)	2 rates (IH, CH) x 2 explainations (HR group: explained as heartbeat sounds, noise group: explained as noise)	Image ratings about discomfort; overall unpleasantness ratings	GSR	Unpleasant level: significantly higher in HR and IH group than in other three groups (*p* < 0.05)	GSR: (1) noise group was significantly lower than HR group (*p* < 0.01); (2) CH condition was significantly lower than IH condition (*p* < 0.05)
Hirschman et al. (1977)	2 rates (IH, CH) x 2 explainations (HR group: explained as heartbeat sounds, ES group: explained as as extraneous sounds)	Image ratings about lust;	GSR	Lust level: (1) no significant difference between HR and ES groups; (2) CH condition was significantly lower than IH condition (I < 0.05)	GSR: within HR group, CH condition was significantly lower than IH condition (I < 0.05)
Makkar and Grisham (2013)	IH, DH	PANAS, BQ, FAQ, SCQ, TQ	HR	Anxiety level: DH condition was significantly lower than IH condition (p < 0.05)	No significant difference among feedback conditions
Menyhart and Gleary (1986)	3 rates (fast IH: from 60 to 140 bpm, medium IH: from 70 to 120 bpm, slow IH: from 80 to 100 bpm) x 2 volumes (loud: 86db, soft: 55 db)	SAQ, FSS, APQ	HR	Anxiety level: slow/soft and fast/loud conditions were significantly lower than slow/loud and fast/soft conditions (*p* < 0.01)	No significant difference among feedback conditions
Misovich (1974)	GSR change condition: feedback indicating a substantial change in GSR; no GSR change condition: feedback indicating no change in GSR.	Image ratings about lust;	/	Lust level: no GSR change condition was significantly lower than GSR change condition (*p* < 0.01)	/
Pan et al. (2020)	TH, NF	Subjective self-rating:−5 (negative)~+5 (positive)	SCL, ST, HR	Positive level: NF condition was significantly lower than TH condition (*p* < 0.01)	ST: Higher ST in TH condition than NF condition (*p* < 0.05)
Parkinson and Manstead (1986)	2 rates (IH, CH) x 2 instructions (instruct to attend, instruct to ignore) x 2 explainations (explained as SCL feedback, explained as neutral sound) x 2 sound tracks (sound track: pulsed, tone)	Image ratings about lust;	/	Lust level: CH condition was significantly lower than IH condition (*p* < 0.001)	/
Parkinson and Colgan (1988)	2 rates (increasing SCL, constant SCL) x 2 instructions (instruct to attend, instruct to ignore) x 2 explainations (explained as SCL feedback, explained as neutral sound)	Image ratings about pleasantness	/	Positive level (positive images): constant SCL condition was significantly lower than increasing SCL condition (*p* < 0.05) Negative level (negetive images): increasing SCL condition was significantly lower than constant SCL condition (*p* < 0.025) (only when subjects told to ignore neutrally described sounds)	/
Rohrmann et al. (1999)	NF, arousing feedback, reassuring feedback	State Trait Anxiety Inventory (STAI), respective item selected from the modified version of the adjective checklist on emotions (EWL)	HR, GSR, BP, Cortisol	Anxiety level: reassuring feedback condition was significantly lower than NF condition, NF condition was significantly lower than arousing feedback condition (*p* = 0.07)	HR change/BP change/GSR change/Cortisol change: NF condition was lower than arousing feedback, arousing feedback was lower than reassuring feedback (only heart rate yields a significant effect *p* = 0.03)
Shahidi and Baluch (1991)	NF, IH, DH	Ratings about embarrassment	/	Anxiety level: DH condition was significantly lower than NF condition, NF condition was significantly lower than IH condition (*p* < 0.01)	/
Stern et al. (1972)	Exp1: 3 rates (IH, DH, CH) x 2 explainations (HR groups: explained as heartbeat sounds; ES group: explained as extraneous sounds) Exp3: 3 rates (CH, IH, DH) x 3 explainations (HR group: explained as heartbeat sounds, ES group: explained as as extraneous sounds, ES-T-I group: explained as extraneous sounds and instruct to attend)	Image ratings about lust; image ratings about unpleasant	HR, GSR	lust level: (1) no significant difference between conditions in ES group; (2) within HR group, CH condition was significantly lower than IH condition (*p* < 0.01); (3) within ES-T-I group, CH condition was significantly lower than IH condition (*p* < 0.05)	HR (lust): ES group was significantly lower than HR group (*p* < 0.05) GSR change (lust): (1) ES group was significantly lower than HR group; (2) within HR group, DH condition was lower than IH condition HR (unpleasant): ES group was significantly lower than HR group (*p* < 0.05)
Tajadura-Jimenez et al. (2008)	2 rates (60 bpm CH, 110 bpm CH) x 2 (with vibration, without vibration), NF	SAM, free-recall task	HR	Arousal ratings: 110 bpm CH condition was significantly lower than 60 bpm CH condition (*p* < 0.05)	HR: NF condition was significantly lower than other conditions (*p* < 0.05)
Telch et al. (2000)	TH, 90 bpm paced tone sounds, NF	Subjective fear scale, Coping self-efficacy scale, Threat expectancies scale	HR	Fear level: TH condition was significantly lower than other conditions at post-treatment (*p* < 0.01)	No significant difference among feedback conditions
Thornton and Hagan (1976)	NF, IH, CH	Image ratings about unpleasant	HR	Unpleasant level: CH condition was significantly lower than IH condition (*p* < 0.003)	No significant difference among feedback conditions
Valins (1966)	3 rates (IH, DH, CH) x 2 explainations (HR group: explained as heartbeat sounds; noise group: explained as noise)	Image ratings about lust; image choices; Delayed image rankings	/	Lust level: (1) noise group was significantly lower than HR group (*p* < 0.05); (2) DH condition was significantly lower than IH condition (*p* < 0.05)	/
Wang et al. (2021)	Exp1: TH, NF Exp2: fake heartbeat feedback (didn't explain its frequencies), NF	Subjective emotion self-ratings (happiness, sadness, fear, anxious, and anger)	SCR, ST, HR, RR	Happiness ratings: (1) NF condition was significantly lower than TH condition (*p* = 0.28); (2) within positive music, NF condition was significantly lower than fake heartbeat condition (*p* < 0.01) Anxiety ratings: within neutral music, NF condition was significantly lower than fake heartbeat condition (*p* < 0.011)	SCR: NF condition was significantly lower than TH condition (*p* = 0.021) HR: NF condition was significantly lower than TH condition (p = 0.003) HR: NF condition was significantly lower than TH condition (*p* < 0.001)
Young et al. (1982)	IH, DH	Verbal reports of discomfort	HR	Unpleasant level: DH condition was significantly lower than IH condition (*p* < 0.01)	HR: DH condition was significantly lower than IH condition (*p* < 0.001)

CH, Constant heartbeat feedback; IH, Increasing heartbeat feedback; DH, Decreasing heartbeat feedback; TH, True heartbeat feedback; NF, No feedback; BP, Blood pressure; GSR, Galvanic Skin Response; HR, Heart Rate; RESP, Respiration; ST, Skin Temperature; APQ, Autonomic Perception Questionnaire; APQ, Autonomic Perception Questionnaire; BAS, Behavioral Activation System; BFNE, Brief Fear of Negative Evaluation Scale; BIS, Behavioral Inhibition System; BQ, Behavior Questionnaire; CASI, Childhood Anxiety Sensitivity Index; DASS, Depression Anxiety Stress Scales; EAQ, Emotion Awareness Questionnaire; ECQ, Emotion Control Questionnaire; ERQ, Emotion Regulation Questionnaire; FAQ, Focus of Attention Questionnaire; FSS, Fear Survey Schedule; MAIA, Multidimensional Assessment of Interoceptive Awareness; PANAS, Positive and Negative Affect Scales; SAM, Self-Assessment Manikin; SAQ, State Anxiety Questionnaire; SCARED, Screen for Child Anxiety Related Emotional Disorders; SCQ, Social Cognitions Questionnaire; SPS, Social Phobia Scale; STAI, State-Trait Anxiety Inventory; TAS, Toronto Alexithymia Scale; TQ, Thoughts Questionnaire.

### 3.4. Descriptions and main outcomes of the included studies

Based on our preliminary analysis, we found that these studies were conducted with diverse target emotions, employing different modalities and content of feedback, as well as varying interpretations of the feedback signals before their delivery to the subjects. And we noticed that these distinct factors play crucial roles in influencing the effectiveness of physiological feedback technology for ER.

Here we provided concise descriptions of the experimental overview, results, and key conclusions of these studies, along with brief comparative analyses of studies with similar experimental designs but divergent findings. And the descriptions are organized into two subsections, focusing on two distinct aspects of these studies. The first subsection concentrates on the influence of feedback modality, feedback content, and target emotion on the intervention effects. Within this subsection, we initially categorize the studies into two groups based on the modality used, namely heart rate modality and other modalities, and subsequently present the findings according to the categories of target emotions. The second subsection centers on the impact of different interpretations of the feedback signals on the intervention effects. Given the limited number of studies and the clear directionality of the conclusions in this regard, we classify the studies based on their conclusions and present them accordingly.

#### 3.4.1. Modality and content of feedback

##### 3.4.1.1. HR modality

In total, 22 studies provided participants with heartbeat feedback during emotion elicitation to examine the effectiveness of this modality for ER and further investigate the effect caused by the content of the feedback.

These studies were first conducted in lust, to ascertain whether the labeling of emotional stimuli would be affected by information concerning internal reactions. Valins used images of seminude females to elicit lust emotion in male subjects, along with playing sounds that were allegedly their heartbeats (Valins, 1966). These sounds were pre-recorded audio tapes of tones at different frequencies, creating three different kinds of heartbeat feedback conditions (CH, IH, and DH). The results of the subjective rating for lust showed a significant difference among the three conditions: the IH condition had the highest lust level, the DH condition had the second highest lust level, and the CH condition had the lowest lust level.

To further validate the results of Valins, Stern et al. (1972) and Botto et al. (1974) performed an experiment similar to Valins' experiment. The subjective ratings in Botto et al. showed consistent results with Valins' for the three heartbeat feedback conditions. However, Stern et al. showed that the IH condition caused the highest ratings, the DH condition caused the lowest ratings, and the CH condition had ratings intermediate between these two conditions, which was not entirely consistent with the study by Valins. The inconsistency between the results of the above three studies lies in the comparison of DH and CH conditions. However, although the scores generated by these two conditions were different between these three studies, the differences were not significant.

Three more studies replicated Valins' experimental design to explore the effects of the frequencies of heartbeat feedback on lust regulation, but these studies included only the CH and IH conditions (Goldstein et al., 1972; Hirschman et al., 1977; Parkinson and Manstead, 1986). The results of these studies showed a significant difference between feedback conditions on lust level, and the IH condition led to a significantly higher subjective rating for lust compared to the CH condition, which confirmed Valins' conclusion.

A series of studies investigated physiological feedback in anxiety regulation. To assess the ER effect of heartbeat feedback in actual anxiety situations, in the study conducted by Borkovec et al. (1974), speech-anxious subjects were exposed to 1 of 5 feedback conditions (CH, IH, DH, and two control conditions) during the second of three consecutive speeches. Their results showed no significant differences in any measure among the feedback conditions during the feedback speech, but post-test speech measures revealed that the DH and CH conditions showed significantly lower anxiety levels than the IH condition.

In another study that investigated the effects of heartbeat feedback on anxiety, dentally anxious subjects viewed a videotape of a provocative dental procedure, while they were exposed to IH or DH condition (Young et al., 1982). Their results showed that male subjects in the DH condition responded to the video with less unpleasantness than those in the IH condition, but no comparable effect was found for females. They also found a tendency for the feedback conditions to interact with gender, which seems to imply a potential correlation between physiological feedback effects and gender.

Additionally, to investigate the effect of heartbeat feedback on speech anxiety, Shahidi and Baluch recruited subjects to speak in front of a camera, and after that, they presented some subjects with the heartbeat sound feedback (IH or DH) while showing them a replay video of their performance during the speech (Shahidi and Baluch, 1991). In their results, the anxiety levels were significantly higher in the IH condition and lower in the DH condition compared to the non-feedback condition.

In one study, researchers designed a virtual reality scene of being suddenly surrounded by smoke during a fire evacuation of a building to induce an anxiety experience, during which some subjects were subjected to one of the feedback conditions (Health Bar condition: a horizontal green bar showed the level of health of the user's avatar during the virtual experience; FPS condition: exploited the aversive auditory and visual stimuli to indicate that the user's avatar is getting hurt; bioFPS condition: identical to the FPS condition except for the fact that the heartbeat sound was related to the participants' actual HR) (Chittaro, 2012, 2014). Larger pre-post differences showed that the bioFPS condition caused a higher anxiety level compared to the other two conditions, indicating a better ER effect of personalized feedback materials based on actual physiological information.

Makkar and Grisham used a richer form of heartbeat feedback in their study, in which subjects not only heard the audio feedback of their heartbeat but also observed HR values and ECG waveforms on a screen (Makkar and Grisham, 2013). The results of this study also showed that the IH condition caused a significantly higher level of anxiety compared to the DH condition.

A study used vibration to provide feedback heartbeat to the subjects while eliciting anxiety, and among their four conditions (NF, TH, 60bpmCH, and vibration condition: 60bpm vibration without explanation), the 60bpmCH condition had significantly lower anxiety levels than the NF and TH conditions (Costa et al., 2016).

Another study also provided heartbeat feedback with vibrations and the subjects were notified that they were about to participate in a public speaking task (Azevedo et al., 2017). During preparation for the speech, some subjects received heartbeat feedback that was 20% slower than their actual heart rate at baseline, while other subjects in the control group did not receive such feedback. They found that the presence of the feedback, as opposed to its absence, had a significant calming effect on participants' anxiety and caused significantly lower subjective anxiety levels and Skin conductance levels (SCL).

In addition to studies on anxiety, a series of studies has been conducted on fear. Menyhart and Gleary recruited subjects with different levels of height fear to participate in the task of walking toward the edge of a flat roof area 20 m above ground level to elicit their fear (Menyhart and Gleary, 1986). There were three feedback rates (fast IH: from 60 to 140 bpm, medium IH: from 70 to 120 bpm, slow IH: from 80 to 100 bpm) crossed with two feedback volumes (loud: 86 db and soft: 55 db), making a total of six experimental conditions. The results of this study showed a significant interaction between the rate and volume of the heartbeat feedback, with slow/loud and fast/soft conditions resulting in significantly higher anxiety levels than the slow/soft and fast/loud conditions, but the influence of each of the two variables on the fear level was rather insignificant.

To investigate the effect of heartbeat feedback on claustrophobic fear, Telch et al. recruited a group of non-clinical students who showed marked fear of claustrophobia and instructed them to receive a 30-min self-directed exposure to a claustrophobic chamber (Telch et al., 2000). During the exposure, one of three conditions (TH, 90 bpm paced tone sounds, and NF) were provided in six 5-min exposure trials. Their results showed that the participants in the TH condition had significantly lower fear levels after the intervention, and a higher percentage of participants fulfilled the criteria for clinically significant change at post-intervention, compared to the participants in the other two conditions.

Three other studies investigated the role of heartbeat feedback in the regulation of unhappiness. They used images of people who were badly injured or those who died in car accidents (Stern et al., 1972), images of people who died violently (Hirschman, 1975; Hirschman and Hawk, 1978), or images of skin disease (Thornton and Hagan, 1976) to elicit negative emotion (unpleasantness) in subjects. In these three studies, the unpleasant ratings of the images showed that, among the CH, DH, and IH conditions, the IH condition resulted in a significantly higher unpleasantness level compared to the other two conditions.

Several studies have investigated the regulatory effects of physiological feedback on non-specific positive, neutral, and negative emotions. Two studies have investigated the effect of heartbeat feedback on positive and neutral emotions (Pan et al., 2020; Wang et al., 2020). Both studies achieved TH by instructing subjects to place their palms on their chests to feel their heartbeats, and the results of both studies showed that the presence of physiological feedback significantly increased subjects' ratings of positivity or happiness with music compared to its absence.

Another study explored the effects of different rates of heartbeat feedback (60 bpm CH and 110 bpm CH) and the presence or absence of vibration feedback (with and without vibration) on positive and negative emotions (Tajadura-Jimenez et al., 2008). The emotions are elicited by positive and negative images from IAPS (5 on a 9-point arousal scale; 3 and 7 on a 9-point valence scale for negative and positive images, respectively). The results of the positive and negative emotion ratings showed that the increase in heart rate significantly increased arousal ratings, but no significant effect was found for valence ratings. Although the effect of vibration alone did not reach significance, the presence of vibrations increased the difference between the two feedback rates.

Finally, one study used five virtual environment scenarios to induce five different emotions (happiness, anxiety, fear, disgust, and sadness), and the results of the emotional scales after each scenario were averaged and evaluated (Dey et al., 2018). During the experiment, they manipulated the feedback in five ways: in the two DH conditions, the frequency of subjects' heartbeats was decreased by 30% and 15%; in the two IH conditions, it was increased by 30% and 15%; and in the TH condition, it was provided the feedback as is. The results showed that −15%DH, −30%DH, and +15%IH heartbeat feedback increased positive emotions (interest and excitement), whereas +15%IH heartbeat feedback increased negative emotions (scariness, nervousness, and fear). Such results deviate significantly as compared to those of other studies, which is very likely caused by the different effects of feedback on different emotions, and the average ratings against different emotional outcomes lead to confounding between effects.

##### 3.4.1.2. Other modalities

Besides the heartbeat, some studies realized physiological feedback with other modalities. One study used GSR as a modality to examine the effect of this kind of physiological feedback on general internal arousal during emotional and non-emotional stimulation (Misovich, 1974). For this study, we focused only on the conditions with emotional stimulation. Similar to the previous studies, Misovich elicited lust in male subjects by showing them images of naked women, while all of the subjects received made-up feedback through a GSR meter box indicating that their GSRs had significantly increased in response to five of the slides and remained unaffected by the other five. From the results of this study, we found that increased GSR readings elicit greater lust levels compared to unaffected GSR readings, which is consistent with the effects of the heartbeat feedback.

In Parkinson et al.'s study, both positive and negative emotions were elicited to explore the influence of GSR feedback on these emotions (Parkinson and Colgan, 1988). They used images of animals (the negative images depicted various unpleasant insects, spiders, and snakes, while the positive images were of kittens and other “cute” animals) as emotional materials, and with these images, a continuous tone whose changing loudness reflected SRL variation was played. Their results showed that positive images were rated as more pleasant when coupled with the IH condition than when coupled with the CH condition; negative images were rated as less pleasant when coupled with the IH condition than when coupled with the CH condition.

Ehlers et al. applied GSR and pupil size as feedback modalities to observe how this feedback affect positive and negative emotions elicited by sounds from IAD-2 (higher than 7 on a 9-point arousal scale and lower than 3 and higher than 7 on a 9-point valence scale for negative and positive sounds, respectively) (Ehlers et al., 2021). Pupil size feedback was applied to both positive and negative emotions, and GSR feedback was applied only to negative emotions. Their results showed that weak, but context-sensitive, GSR feedback reduced the actual emotional physiological response to negative emotions; but pupil size feedback did not show the expected ER effect, which is probably due to the unsimplified and ambiguous nature of the pupil signal, making it difficult for subjects to understand the emotional meaning represented by the feedback signal promptly.

Finally, one study aimed at regulating public speaking anxiety by providing verbal comments about the participants' physiological arousal state (physiologically aroused/physiologically relaxed) during the anticipation of public speaking (Rohrmann et al., 1999). There were three feedback conditions: NF condition; arousing feedback condition: participants were told that they are physiologically aroused and nervous; reassuring feedback condition: participants were told that they are physiologically calm and relaxed. Within the three conditions, anxiety levels were significantly higher in the arousing feedback condition and lower in the reassuring feedback condition.

#### 3.4.2. Explanation of feedback

In addition to examining the role of feedback modalities and content in ER, there are several studies that have aimed to answer the question about how the explanation of feedback affects the effects of this intervention.

Three studies showed that the ER effect was contingent on the interpretation given to the feedback. Explaining the feedback content as the subjects' own physiological information could enhance the ER effect of this intervention. In Parkinson et al.'s study mentioned previously, the authors also found that within the heart-rate condition, those subjects who were told to pay attention to the feedback showed greater rating differences between these two conditions than those who were told to ignore it. To test the hypothesis that if a subject is led to believe that a feared object does not affect him internally, he or she will show less fear toward that object. Borkovec and Glasgow (1973) performed a similar experiment. During this experiment, two groups of participants were exposed to slides of snakes and slides of the word “shock,” paired with mild shock. The experimental participants heard the feedback of heartbeat sounds, implying that they did not respond to the snake images but responded to the shock images. The control subjects heard the same feedback but were told that the sounds were extraneous noises. The results of the post-experimental live snake exposure showed that the former group was less fearful of snakes, implying that the explanation of the feedback influenced the ER effect, and explaining the feedback content as physiological information of the subjects enhanced the effect of the feedback. In Hirschman et al. study (Hirschman, 1975; Hirschman and Hawk, 1978), the two IH conditions (with or without interpretation) had differences in terms of the presence of interpretation caused by a more significant effect of enhancing unpleasant feelings and demonstrating the critical role of the interpretation in physiological feedback.

Two studies have shown that this explanation not only enhances the corresponding regulatory effect but is also a prerequisite for this effect to work and that this intervention can only be effective if the subjects interpret the received feedback as their own physiological information. Besides examining the role of frequency of heartbeat feedback in lust regulation, Valins' study also explored how the explanation of feedback influenced the regulatory effects. During viewing the images mentioned above, some subjects were led to believe that they were hearing an amplified version of their hearts beating, while others heard identical sounds but did not associate them with their own heartbeats. The comparison of the results of these two groups showed that when the sounds were not considered heartbeats, they had virtually no effect on the subjects' ratings. The results of the study conducted by Costa et al. showed that between the two CH conditions (with or without interpretation), only the condition with interpretation kept the anxiety of the individuals at low levels.

However, in the other two studies, the explanation failed to show such an effect. While the presence of the explanation in the study conducted by Hirschman et al. mentioned above served to enhance the effect, this effect was not observed in the other study conducted by Hirschman et al. By comparing these two studies, we found that the differences between them were mainly in emotion type and participant gender. The study that evaluated unpleasantness in females showed a beneficial effect of explanation, whereas the study that evaluated lust in males failed to show such an effect. Similarly, although the significant contribution of interpretation has been shown in another of Parkinson et al.'s studies using heartbeat as a modality, as mentioned above, a similar contribution was not observed in their other study using GSR as a modality. In the other study, they used a continuous-tone soundtrack that increased in pitch as the feedback of GSR and discovered that the effects of physiological feedback do not always depend upon the meaning, given the sounds are not directly related to subjects' attention level, and significant rating effects were obtained only for groups instructed to ignore the auditory stimulus when it was interpreted as a neutral sound rather than as feedback. Modality, as the significant difference in these two studies, may be the reason for such different results, but there were also other significant differences between these studies such as emotion type and gender composition, making it difficult for us to conclude exactly what factors were altered to account for this difference in results.

## 4. Discussion

To clarify the effectiveness of physiological feedback as an ER method to regulate ongoing emotions, we performed a systematic literature search, which yielded 27 relevant articles (25 studies). Based on these studies, we concluded the influence of physiological feedback on ongoing emotional processes, as well as summarized the factors that influence its effectiveness. In addition, we explored the optimal use of this intervention to realize the intended ER goals.

### 4.1. Effect of physiological feedback

Among the included studies, all 25 of the studies showed a regulatory role of physiological feedback in different emotions.

When applied to lust, comparison among the different feedback conditions showed that IH can cause significantly higher subjective lust levels compared to CH and DH conditions (Valins, 1966; Goldstein et al., 1972; Stern et al., 1972; Botto et al., 1974; Hirschman et al., 1977; Parkinson and Manstead, 1986); heartbeat feedback and GSR feedback with changing values can also cause a higher lust level compared to that with constant values (Misovich, 1974).

When applied to anxiety, a comparison between the feedback and non-feedback conditions showed that IH has a significant effect on increasing participants' anxiety levels, while DH or low-rate CH can significantly reduce their anxiety (Borkovec et al., 1974; Young et al., 1982; Shahidi and Baluch, 1991; Chittaro, 2012, 2014; Makkar and Grisham, 2013; Costa et al., 2016; Azevedo et al., 2017); heartbeat feedback and verbal comments about physiological arousal state (physiologically aroused or physiologically relaxed) can also influence participants' anxiety level, with the anxiety level being significantly higher in the arousing feedback condition and lower in the reassuring feedback condition (Rohrmann et al., 1999).

When applied to fear, a comparison between the feedback and non-feedback conditions showed that TH can significantly reduce subjects' fear levels after the feedback intervention. However, since there are few fear-related studies, no study has identified the role that physiological feedback plays as a real-time intervention in the fear elicitation process; thus further research is needed (Menyhart and Gleary, 1986; Telch et al., 2000).

When applied to unhappiness, comparison among the different feedback conditions showed that IH can result in a significantly higher unpleasantness level compared to CH and DH conditions (Stern et al., 1972; Hirschman, 1975; Thornton and Hagan, 1976; Hirschman and Hawk, 1978).

When applied to non-specific emotions (positive, negative, and neutral emotions), a comparison between the feedback conditions and non-feedback condition showed that TH can cause a higher pleasantness level in positive emotion; and comparison among the different feedback conditions showed that IH and increasing SCL conditions can cause a higher pleasantness level in positive emotion (Parkinson and Colgan, 1988; Tajadura-Jimenez et al., 2008; Pan et al., 2020; Wang et al., 2020; Ehlers et al., 2021).

Globally, this intervention appears to be an effective ER method for different emotions. In the studies mentioned above, it achieved the upregulation or downregulation of different emotions according to the regulation goals.

### 4.2. Factors that influence the effectiveness of physiological feedback

With the ER ability of physiological feedback being well-established, we further comprehensively analyzed the included studies and summarized several factors that influence the outcomes of this intervention. Considering these factors enables this technology to achieve its optimal ER effect.

#### 4.2.1. Content of physiological feedback

First, the content of feedback (i.e., the emotional physiological information it conveys) has a decisive influence on the regulatory effect, which determines the regulation's direction.

According to the attribution or cognitive-arousal theory of emotional experience, the visceral-autonomic nervous system feedback and the cognitive interpretation of the stimulus that induced this visceral activation determine the emotional experience, and the provision of the physiological feedback affected the cognitive interpretation of the feedback from the visceral-autonomic nervous system, thus altering the emotional experience. As a result, the content of the physiological feedback determines its ER effect.

In our review, all of the studies showed that feedback implying strong physiological arousal (strong feedback) (i.e., heartbeat feedback with higher rates, heartbeat feedback with increasing rates, GSR feedback with increasing values, and verbal comments of increased physiological arousal) tends to upregulate the emotion, regardless of the category of the emotion.

For feedback implying weak physiological arousal (weak feedback) (i.e., heartbeat feedback with slow frequency, heartbeat feedback with decreasing frequency, GSR feedback with decreasing value tend, and verbal comments of decreased physiological arousal), some studies have reported that weak feedback can downregulate different emotions; however, consistent conclusions cannot always be drawn. Meanwhile, although weak feedback was included as an experimental condition in 13 studies in total, few of the remaining studies designed a non-feedback condition in their experiments, making it difficult for us to generalize the specific effects of this feedback. Therefore, whether weak feedback can provide a downregulatory effect on emotions remains to be further investigated.

In addition to the strong and weak feedback mentioned above, there is also feedback that reflects the actual physiological state of the subjects (true feedback). Among our included studies, one study showed that the presence of true feedback did not affect subjects' anxiety. In two other studies, true feedback resulted in an upregulation of positive emotions; another study showed that true feedback downregulated subjects' fear. Based on these results, it appears that the ER effect of true feedback does not have a significant pattern, and in our opinion, this may be because unlike the strong or weak feedback, true feedback does not convey directionally distinct information about subjects' own physiological state, which makes it more difficult for such feedback to influence subjects at the cognitive level and change their emotional feelings.

#### 4.2.2. Explanation of physiological feedback

Second, the explanation of feedback is also a critical factor of this intervention, which significantly influences its credibility and thereby determines the regulation's effectiveness.

The credibility of the physiological feedback refers to the subjects' conviction that the feedback provided is derived from their own physiological signals, which determines whether the emotional physiological information conveyed by the feedback is functional, and the experimenter's explanation of the feedback before providing it undoubtedly affects this credibility enormously.

Many studies have investigated the influence of explanation on physiological feedback' ER effect. Although the absence of the explanation did not influence the results in two of these studies, in the majority of the studies, this explanation has been proven to be a key factor in this intervention.

Three studies have shown that the ER effect was contingent on the interpretation given to the feedback, and explaining the feedback content as the subjects' own physiological information could enhance the ER effect of this intervention.

Two other studies showed that this explanation not only enhances the corresponding regulatory effect but is also a prerequisite for this effect to work. Namely, this intervention is only effective if the subjects interpret the received feedback as their own physiological information. For this reason, it is essential to interpret the feedback as the subject's own physiological signal presentation and to make the subject fully aware of the association between the feedback signal and the emotional status before providing the feedback.

#### 4.2.3. Authenticity and real-time capability of physiological feedback

Third, the authenticity and real-time capability of the feedback affect the credibility of the feedback, as well as the accuracy of the emotional physiological information conveyed by the feedback, thus influencing the effect of regulation.

Whether the feedback is based on the real physiological state of the participants (authenticity) and whether it can be adjusted to their changing physiological state in real-time (real-time capability) also influence the ER effect of the physiological feedback. Although it was mentioned above that strong feedback can facilitate emotion elicitation and serve to upregulate emotions, this does not mean that the more dramatic the physiological response is, the more significant the upregulation effect will be. When the physiological feedback is exaggerated, participants are more likely to realize the manipulation of the feedback and the irrelevance between the received feedback and their actual physiological state, making the feedback lose its credibility and failing to achieve the expected ER effect. In fact, the best way to avoid this situation is to provide feedback with authenticity and real-time capability, which enables the feedback signal to change based on the subjects' actual physiological state, preventing overly exaggerated physiological feedback under reasonable manipulation.

Other than ensuring the credibility of the feedback, the authenticity and real-time capability of the feedback can also guarantee the accuracy of the emotional physiological information conveyed by the feedback, which is because the physiological feedback that unrelated to the subject's true physiological state or that does not have real-time capabilities may have different effects on different individuals under the same conditions. For example, heartbeat feedback at 70 bpm can be considered weak feedback for subjects with HR around 80 bpm, while the same feedback can be considered as strong feedback for subjects with HR around 60 bpm. Similarly, it is unclear whether increasing heartbeat feedback whose frequency increased from 60 bpm to 80 bpm should be regarded as strong feedback that facilitates emotion elicitation for an individual whose heart rate changed from 60 bpm to 90 bpm during this period. These examples above illustrate the importance of authenticity and real-time capability of feedback, as evidenced in a study whose emotion regulation goal was to upregulate anxiety, the results showed that the IH condition related to individuals' actual HR caused a higher anxiety level compared to the other IH condition.

#### 4.2.4. Modality of physiological feedback

Fourth, the modality of the feedback determines how well the individuals understand the emotional and physiological information conveyed by the feedback, thus influencing the regulatory effect.

When using physiological feedback to regulate emotions, the selection of the feedback modality largely affects the effectiveness of the feedback. The physiological parameters used as the feedback modality should be relevant to emotions and its changes should reflect the changes in emotions. Meanwhile, its relevance and changes could be perceived and understood by individuals. The choice of feedback modality may influence the participants' interpretation of their own emotional physiological information, so as to achieve the purpose of ER.

Of all the modalities, the heartbeat has been the most studied due to the simplicity of its implementation technique and the clarity of the information it expresses. The majority of these studies have achieved significant ER effects.

In addition to the heartbeat modality, GSR was used as a feedback modality in three studies, the general physiological state was used in one study, and pupil size was used in one study. Among these modalities, both GSR and general physiological state have been shown to be effective for ER, and these two modalities can achieve similar ER outcomes as heartbeat feedback, whereas pupil size feedback failed to show the expected ER effect, which is probably due to the unsimplified and ambiguous nature of the pupil signal, making it difficult for subjects to understand the emotional meaning represented by the feedback signal.

Overall, the heartbeat is a common feedback modality that has been well-established for its significant ER capacity. Since few studies have been conducted on modalities other than the heartbeat, the effectiveness of these modalities in ER remains to be further confirmed.

### 4.3. Limitations

While all of these findings are encouraging regarding the ER potential of physiological feedback interventions, it is difficult to ignore that these studies have several limitations. Here, we will specify these limitations.

(1) Samples: first, most studies had limited sample sizes, and few used randomization to select their samples; second, with the results of multiple studies reflecting the potential gender differences in such interventions, there were several studies with significant gender bias. These limitations may lead to biased results, making the conclusions drawn from individual studies difficult to generalize.

(2) Experimental design: the majority of these studies did not include a non-feedback control condition in their experiments, and the conclusions can only be drawn by comparing the differences between the experimental conditions. Not only does this make it impossible to determine the specific regulatory effect of each feedback condition in a single study, but it also makes the results less comparable between studies.

(3) Interventions: first, the emotional elicitation materials used in many studies were self-selected, non-standardized materials, while very few studies have examined the validity of these materials. Thus, there is no assurance either of the targeted emotional intensity arousals in the subjects or of whether the target emotion was elicited rather than other emotions. These uncertainties prevent the results of these studies from accurately representing the effects of physiological feedback on such emotions, which may also explain the inconsistent results between some of the studies. Second, the majority of studies have not considered the authenticity and real-time capability of physiological feedback, which likely resulted in different regulatory effects of the same feedback condition in different subjects. As mentioned above, these factors affect the credibility of the feedback and the accuracy of the physiological information about emotions conveyed by the feedback, and neglect of these factors probably weakens or even alters the overall regulatory effect of physiological feedback.

(4) Measurement and evaluation: first, measurement criteria varied between studies, and some studies assessed the effects of feedback specifically for each emotion, while other studies averaged the results of several emotions before analyzing them. Second, in many studies, only psychological assessment instruments were used to measure the effects of physiological feedback, and physiological measurements were not performed simultaneously. Meanwhile, in studies that used physiological parameter measurements, the analysis methods for physiological parameters were rather simplistic. These factors make it difficult to make a conclusion regarding any specific ER effects in different emotions from these results, as well as make the results less comparable between studies and prevent further exploration of the physiological basis of the technology.

(5) Application: As mentioned previously, physiological feedback is a unique real-time emotion regulation technology and its portability is a key factor in putting it into practical application. Only a few studies have considered the portability of this intervention. In most of these studies, physiological feedback related to real physiological states is achieved in the laboratory using mature biofeedback instruments, which are often large and not easily movable.

### 4.4. Implications for future studies

Considering these limitations, future experimental studies should adopt more controlled and better-designed protocols to clearly and effectively confirm the effect of physiological feedback for ER as well as to pave the way for its future practical application.

Future studies should, therefore, (1) include larger sample sizes selected using conventional randomization methods and consider gender differences during subject recruitment and experimental grouping; (2) set reasonable control conditions in experiments to further clarify the effects of feedback; (3) perform robust validity tests on emotion elicitation materials to ensure the applied emotion of physiological feedback, as well as consider the authenticity and real-time capability of the feedback to achieve the physiological feedback as accurately as possible; (4) select more widely applicable and standardized assessment tools, and when available, perform both physiological and psychological analyses; avoid averaging the results of different emotions directly before analysis and perform more in-depth analysis of the physiological results; (5) consider in detail the portability of the technology and test the ER effect of the technology in more practical scenarios.

## 5. Conclusions

This systematic review presents robust evidence affirming the efficacy of physiological feedback technology in the context of ER. Furthermore, we provide a comprehensive overview of key factors that play a crucial role in shaping the outcomes of the intervention, including the content, explanation, authenticity, real-time capability, and modality of physiological feedback. Thoroughly considering the multifaceted factors enables this technology to attain its utmost effectiveness.

The methodological limitations in the existing studies have been explicitly identified, and these results obtained still need to be validated by more well-designed experimental studies. Meanwhile, in future research, it is imperative to conduct comprehensive investigations into the physiological changes induced by this technology, delve deeper into its intricate physiological mechanisms, and establish a robust theoretical framework to facilitate its practical application.

Finally, the results presented in this systematic review serve as a foundation for further research and application of this technology in the field of ER, offering promising prospects for effective emotion management in diverse scenarios and populations.

## Data availability statement

The original contributions presented in the study are included in the article/Supplementary material, further inquiries can be directed to the corresponding author.

## Author contributions

YS contributed to design of the study and wrote the manuscript. TL contributed to quality assessment. JZ contributed to conception and design of the study. All authors contributed to manuscript revision, read, and approved the submitted version.
