# Supplementary material for: Physiological feedback technology for real-time emotion regulation: a systematic review
Source: Front Psychol. 2023 May 12;14:1182667. doi: 10.3389/fpsyg.2023.1182667 (PMC10213271; doi:10.3389/fpsyg.2023.1182667)
Supplement: Supplementary file 1 [file Data_Sheet_1.docx]

**Appendix A.** Quality Assessment of Reviewed Studies

National Institutes of Health National Heart, Lung and Blood Institute Study Quality Assessment Tool for Observational Cohort and Cross-sectional Studies
**Questions：**

1. Was the research question or objective in this paper clearly stated?

2. Was the study population clearly specified and defined?

3. Was the participation rate of eligible persons at least 50%?

4. Were all the subjects selected or recruited from the same or similar populations (including the same time period)? Were inclusion and exclusion criteria for being in the study prespecified and applied uniformly to all participants?

5. Was a sample size justification, power description, or variance and effect estimates provided?

6. For the analyses in this paper, were the exposure(s) of interest measured prior to the outcome(s) being measured?

*Per guidelines, cross-sectional studies analyses are awarded a “No” for this criterion*
7. Was the timeframe sufficient so that one could reasonably expect to see an association between exposure and outcome if it existed?
*Per guidelines, cross-sectional studies analyses are awarded a “No” for this criterion*

8. For exposures that can vary in amount or level, did the study examine different levels of the exposure as related to the outcome (e.g., categories of exposure, or exposure measured as continuous variable)?

9. Were the exposure measures (independent variables) clearly defined, valid, reliable, and implemented consistently across all study participants?

*Most studies’ independent variables* (rather than any specific exposure) *were compared against this criterion*

10. Was the exposure(s) assessed more than once over time?

11. Were the outcome measures (dependent variables) clearly defined, valid, reliable, and implemented consistently across all study participants?
12. Were the outcome assessors blinded to the exposure status of participants?

13. Was loss to follow-up after baseline 20% or less?

14. Were key potential confounding variables measured and adjusted statistically for their impact on the relationship between exposure(s) and outcome(s)?

**Table A.1.** Quality assessment of included studies using the Quality Assessment Tool for Observational Cohort and Cross-Sectional Studies

| **Study** | **1** | **2** | **3** | **4** | **5** | **6** | **7** | **8** | **9** | **10** | **11** | **12** | **13** | **14** | **Quality Rating** |
| --- | --- | --- | --- | --- | --- | --- | --- | --- | --- | --- | --- | --- | --- | --- | --- |
| Azevedo et al. (2017) | Yes | No | NR | NR | No | No | No | NA | Yes | No | Yes | NR | NA | Yes | Fair |
| Borkovec et al. (1974) | Yes | Yes | NR | Yes | No | No | No | NA | Yes | Yes | Yes | Yes | NA | No | Good |
| Botto et al. (1974) | Yes | Yes | NR | Yes | No | No | No | NA | No | No | No | NR | NA | No | Fair |
| Chittaro (2012, 2014) | Yes | Yes | NR | No | No | No | No | NA | Yes | No | Yes | NR | NA | No | Fair |
| Costa et al. (2016) | Yes | Yes | NR | Yes | No | No | No | NA | Yes | No | Yes | NR | NA | Yes | Good |
| Dey et al. (2018) | Yes | Yes | NR | No | Yes | No | No | Yes | Yes | No | Yes | NR | NA | No | Fair |
| Ehlers et al. (2021) | Yes | No | NR | NR | No | No | No | NA | Yes | Yes | Yes | NR | NA | No | Fair |
| Goldstein et al. (1972) | Yes | Yes | NR | Yes | No | No | No | NA | Yes | No | No | NR | NA | No | Fair |
| Hirschman et al. (1975, 1978) | Yes | Yes | NR | No | No | No | No | NA | Yes | No | No | NR | NA | No | Fair |
| Hirschman et al. (1977) | Yes | Yes | NR | Yes | No | No | No | NA | Yes | No | No | NR | NA | No | Fair |
| Makkar and Grisham (2013) | Yes | Yes | NR | Yes | Yes | No | No | NA | Yes | No | Yes | NR | NA | No | Good |
| Menyhart and Gleary (1986) | Yes | Yes | NR | Yes | No | No | No | Yes | Yes | No | Yes | NR | NA | No | Fair |
| Misovich (1974) | Yes | Yes | NR | Yes | No | No | No | NA | No | No | No | NR | NA | No | Fair |
| Pan et al. (2020) | Yes | Yes | NR | Yes | No | No | No | NA | Yes | No | Yes | NR | NA | No | Fair |
| Parkinson et al. (1986) | Yes | Yes | NR | Yes | No | No | No | NA | No | No | No | NR | NA | No | Fair |
| Parkinson et al. (1988) | Yes | Yes | NR | Yes | No | No | No | NA | No | No | No | NR | NA | No | Fair |
| Rohrmann et al. (1999) | Yes | Yes | NR | No | No | No | No | NA | Yes | No | Yes | NR | NA | Yes | Fair |
| Shahidi and Baluch (1991) | Yes | No | NR | NR | No | No | No | NA | No | No | No | NR | NA | No | Poor |
| Stern et al. (1972) | Yes | Yes | NR | Yes | No | No | No | NA | Yes | No | No | NR | NA | No | Fair |
| Tajadura-Jimenez et al. (2008) | Yes | No | NR | NR | No | No | No | NA | Yes | No | Yes | NR | NA | No | Fair |
| Telch et al. (2000) | Yes | Yes | NR | Yes | No | No | No | NA | Yes | No | Yes | NR | NA | Yes | Good |
| Thornton and Hagan (1976) | Yes | No | NR | NR | No | No | No | NA | Yes | No | No | NR | NA | No | Poor |
| Valins (1966) | Yes | Yes | NR | Yes | No | No | No | NA | No | No | No | NR | Yes | No | Fair |
| Wang et al. (2021) | Yes | Yes | NR | Yes | No | No | No | NA | Yes | No | Yes | NR | NA | No | Fair |
| Young et al. (1982) | Yes | Yes | NR | Yes | No | No | No | NA | No | No | No | NR | Yes | No | Fair |

No, study does not satisfactorily meet question criteria; Yes, study satisfactorily meets question criteria; NA, not applicable; NR, not reported. Questions derived from the National Institutes of Health National Heart, Lung and Blood Institute, Study Quality Assessment Tool for Observational Cohort and Cross-sectional Studies.
